# The Effects of Different Purifying Methods on the Chemical Properties, *in Vitro* Anti-Tumor and Immunomodulatory Activities of *Abrus cantoniensis* Polysaccharide Fractions

**DOI:** 10.3390/ijms17040511

**Published:** 2016-04-06

**Authors:** Shaowei Wu, Xiong Fu, Margaret A. Brennan, Charles S. Brennan, Chen Chun

**Affiliations:** 1 College of Light Industry and Food Sciences, South China University of Technology, Guangzhou 510640, China; zemilym87@yahoo.com (S.W.); cd0328@163.com (C.C.); 2 Centre for Food Research and Innovation, Lincoln University, Lincoln 7648, New Zealand; Margaret.Brennan@Lincoln.ac.nz

**Keywords:** polysaccharide, *Abrus cantoniensis*, purifying method, anti-tumor, immunomodulatory

## Abstract

*Abrus cantoniensis* (Hance) is a popular Chinese vegetable consumed as a beverage, soup or folk medicine. To fully exploit the potential of the polysaccharide in *Abrus cantoniensis*, nine polysaccharide fractions of *Abrus cantoniensis* were isolated and purified (AP-AOH30-1, AP-AOH30-2, AP-AOH80-1, AP-AOH80-2, AP-ACl-1, AP-ACl-2, AP-ACl-3, AP-H and AP-L). Fourier-transform infrared spectroscopy (FT-IR) and gas chromatography (GC) were used to characterize these Abrus polysaccharides fractions (APF). *In vitro* anti-tumor and immunomodulatory activities were also investigated and compared using the rank-sum ratio (RSR) method. Results demonstrated significant differences in the structure and bioactivities among APF, which were associated to the process used for their purification. Among the APF, AP-ACl-3 yield was 613.5 mg/kg of product and consisted of rhamnose (9.8%), arabinose (8.9%), fructose (3.0%), galactose (9.9%), glucose (4.3%), galacturonic acid (3.0%) and glucuronic acid (61.1%) with a molecular weight of 4.4 × 10^4^ Da. Furthermore, AP-ACl-3 exhibited considerable bioactivities significantly preventing the migration of MCF-7 cells and stimulating lymphocyte proliferation along with nitric oxide (NO) production of peritoneal macrophages. AP-ACl-3 could be explored as a novel potential anti-tumor and immunomodulatory agent.

## 1. Introduction

*Abrus cantoniensis* (Hance) belongs to the Abrus genus in the leguminosae family and has been used widely as an edible vegetable and medicinal plant in tropical areas of Asia for thousands of years. The whole plant has been used as a folk medicine for controlling fevers, removing toxicity, preventing jaundice as well as treating infectious hepatitis [1]. Previous studies have also demonstrated the potential biological activities of this plant, including antioxidant [2], anti-tumor [3], antimicrobial [4], hepatoprotective [5], and immunomodulatory activities [6].

In recent decades, numerous polysaccharides isolated from botanical sources such as algae and lichen have been proven to possess a wide range of biological functions such as anti-tumor, antioxidant and immunomodulatory activities [7,8,9]. The exploitation of polysaccharides for pharmaceutical applications has attracted much attention. However, identifying polysaccharides with the desired biological activity is not easy due to the polydispersity of the polysaccharides along with the separation and purification difficulties [10]. These difficulties are especially common in the quest to exploit and extract natural polysaccharides from novel sources. The purity and the amounts of promising polysaccharides are often only available in small quantities for basic structural and biological activities. For instance, many *in vivo* biological studies have been conducted based on non-purified polysaccharides [8,11], or the largest fraction by mass [12], despite the lack of any evidence of their efficacy in relation to bioactivity. Additionally, some polysaccharides have been reported to have little or no bioactivity as a result of unsuitable downstream processing. Thus, exploration of an appropriate purifying process is required for the novel polysaccharides.

To the best of our knowledge, there is a paucity of research available describing the influences of different downstream purification processing techniques on the structure and bioactivities of polysaccharides in *A. cantoniensis*, or fundamental research to explain the underlying mechanisms. The purified polysaccharide can be obtained by various downstream isolation and purification processing, such as ethanol precipitation, membrane separation, fractional precipitation, acidic precipitation with acetic acid, anion-exchange chromatography, gel filtration, and affinity chromatography [13]. Therefore, to fully exploit the potential of the polysaccharide of *A. cantoniensis* and find a suitable purifying process, DEAE-exchange chromatography was combined with stepwise ethanol precipitation and membrane separation leading to the production of nine polysaccharide fractions namely: AP-AOH30-1, AP-AOH30-2, AP-AOH80-1, AP-AOH80-2, AP-ACl-1, AP-ACl-2, AP-ACl-3, AP-H and AP-L. Additionally, the chemical properties and the *in vitro* anti-tumor and immunomodulatory activities of the polysaccharide fractions were evaluated.

## 2. Results

### 2.1. Isolation of Abrus Polysaccharides Fractions (APF)

**Figure 1:**
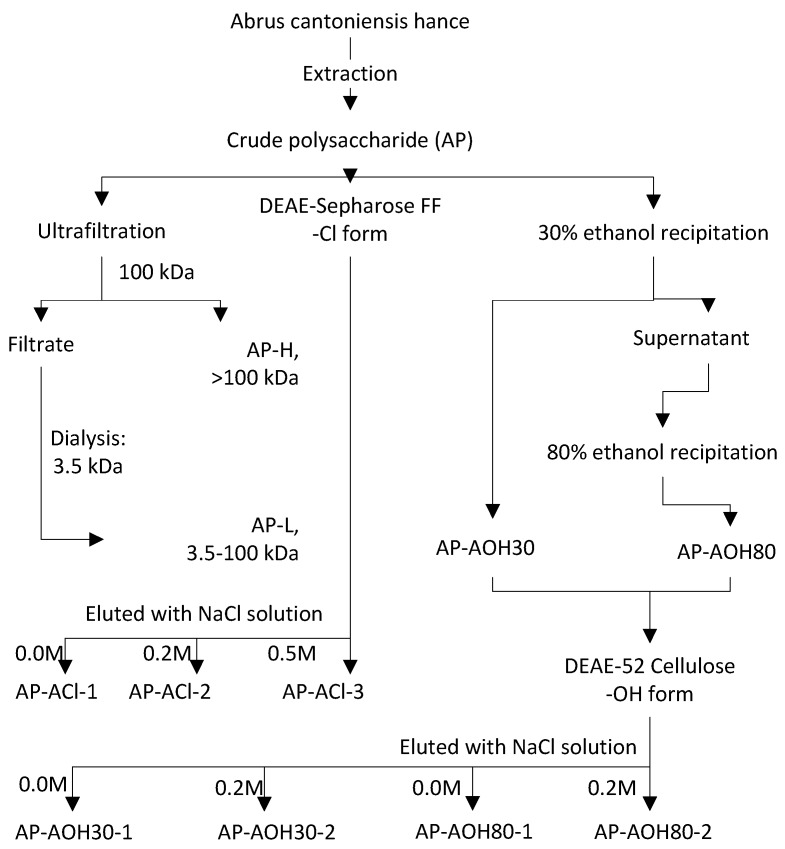
Scheme for isolation and fractionation of polysaccharides from *A. cantoniensis*.

The crude water-soluble polysaccharide was fractionated into nine fractions by different fractionating processes as shown in Figure 1. The elution curves for the purified polysaccharide fractions AP-AOH30-1, AP-AOH30-2, AP-AOH80-1, AP-AOH802, AP-ACl-1, AP-ACl-2 and AP-ACl-3 are given in the supplementary materials Relative yields are described in Table 1. AP-H, AP-ACl-1 and AP-ACl-3 exhibited higher yields than other fractions with the yield of 865.2, 702.3 and 613.5 mg/kg, whereas AP-AOH30-2 had the lowest yield of 64.8 mg/kg. The total carbohydrate content of the isolated polysaccharide fractions ranged from about 55% to 85% determined by phenol-sulfuric acid method. The fractions AP-ACl-1, AP-ACl-2 and AP-ACl-3 each had significantly higher carbohydrate content than other fractions (82.19% ± 2.36%, 84.78% ± 2.28% and 83.93% ± 1.60%, respectively).

### 2.2. Characterization Comparison for APF

#### 2.2.1. Chemical Properties of APF

The uronic acid and sulfate content of APF are also presented in Table 1. AP-OH30-1, AP-OH30-2, AP-ACl-2, AP-ACl-3 and AP-L contained a relatively higher content of uronic acid and sulfate than the other fractions. The uronic acid content in these fractions was 20.33% ± 1.70%, 22.57% ± 1.48%, 10.87% ± 2.01%, 13.62% ± 0.57% and 9.31% ± 1.03%, respectively, and the sulfate content was 8.48% ± 0.43%, 11.05% ± 0.40%, 1.62% ± 0.25%, 3.58% ± 0.16% and 3.88% ± 0.32%, respectively. AP-AOH30-2 contained the highest content of uronic acid and sulfate followed by AP-ACl-3 and the AP-L. Furthermore, four of these five polysaccharides were eluted by NaCl solution, indicating that APF acidic polysaccharides contained more uronic acid and sulfate. No protein was detected by Bradford test in any of the nine fractions. Additionally, the endotoxins levels of all the APF were less than 0.01 EU/mL, indicating that all these polysaccharide solutions were free from endotoxin.

#### 2.2.2. The Distribution of Molecular Weight

The molecular weight distribution shown in Table 1 also demonstrates great differences between the isolated polysaccharide fractions. The fractions AP-AOH80-2, AP-ACl-1, AP-ACl-3 and AP-L were homogeneous, while the other fractions were heterogeneous. Compared with the APF_80%EtOH fractions, APF_30%EtOH fractions had much higher molecular weights, AP-AOH30-1 (11.27 × 10^4^ Da, 62.27%) and AP-AOH30-2 (12.98 × 10^4^ Da, 59.77%), indicating that a lower concentration of ethanol was more effective for precipitating the larger polysaccharides. It was noted that similar polysaccharide fractions were obtained among the APF_30%EtOH and APF_80%EtOH fractions. For instance, both AP-AOH30-1 and AP-AOH30-2 consisted of two polysaccharides of around 1.6 × 10^4^ Da and a larger 10 × 10^4^ Da polysaccharide, but differed in the content of the smaller fraction (15.97% and 34.55%, respectively). Interestingly, no polysaccharide larger than 10 × 10^4^ Da was observed in AP-ACl-1 but existed in AP-ACl-2, suggesting a quite different behavior in the two anion exchange chromatographic purification processes. The larger fraction of AP-H was only 8.54 × 10^4^ Da, indicating that the polysaccharides >10 × 10^4^ Da were lost in the membrane separation. In general, these results demonstrate that the purifying conditions have significant effects on the distribution of molecular weight as well as the content of their sub-fractions.

#### 2.2.3. Fourier-Transform Infrared Spectroscopy (FT-IR) Analysis

The wavelength assignments of APF are depicted in Table 2. The characteristic peaks around 3400 and 2930 cm^−1^ confirmed that all the isolated fractions are polysaccharides. The absorptions for 1743 and 1421 cm^−1^ for AP-AOH30-1, 1743 and 1420 cm^−1^ for AP-AOH30-2, 1735 and 1424 cm^−1^ for AP-ACl-2, 1723 and 1424 cm^−1^ for AP-ACl-3, and 1739 and 1415 cm^−1^ for AP-L are characteristic of a carboxylic group, suggesting the possible presence of uronic acids in these polysaccharides [14,15]. This is in accordance with the results determined by *m*-hydroxydiphenyl method. The bands at around 1240 cm^−1^ are ascribed to the S=O stretching vibration of the sulfate group, while bands at around 580 cm^−1^ are assigned to the bending vibration of O=S=O. Absorptions of around 844 and 890 cm^−1^ are typical for α and β configurations in pyranose form. These differences in characteristic peaks as listed in Table 2 proved that the nine APF were structurally different.

#### 2.2.4. Monosaccharide Composition of APF

As can be seen from Table 3, differences exist in the monosaccharide compositions among APF. The fraction AP-ACl-3 contained GlcA (61.1%), GalA (3.0%), Rha (9.8%), Ara (8.9%), Fru (3.0%), Glc (4.3%) and Gal (9.9%). The fraction AP-AOH30-2 was also found to be rich in GlcA (46.1%), followed by GalA (22.1%), Gal (12.6%), Ara (11.7%), Rha (4.0%) and Glc (3.5%). The lower molecular weight fraction AP-L contained mainly GalA (7.6%), Rha (9.1%), Ara (17.0%), Xyl (3.0%), Fru (5.2%), Glc (30.3%) and Gal (27.7%). All the APF acidic polysaccharides contained GalA and the molar percentage of GalA in AP-AOH30-2, AP-AOH80-2, AP-ACl-2 and AP-ACl-3 were 22.1%, 2.1%, 5.5% and 3.0%, respectively. No acidic sugar was found in AP-AOH80-1 and AP-ACl-1, while AP-AOH30-1 had a considerable amount of GalA (10.0%), suggesting a quite complex behavior existing in the ion-exchange chromatography on the DEAE-52 cellulose column for the AP-AOH30-1.

### 2.3. In Vitro Anti-Tumor Effects of APF

#### 2.3.1. *In Vitro* Cell Proliferation Assay

**Figure 2:**
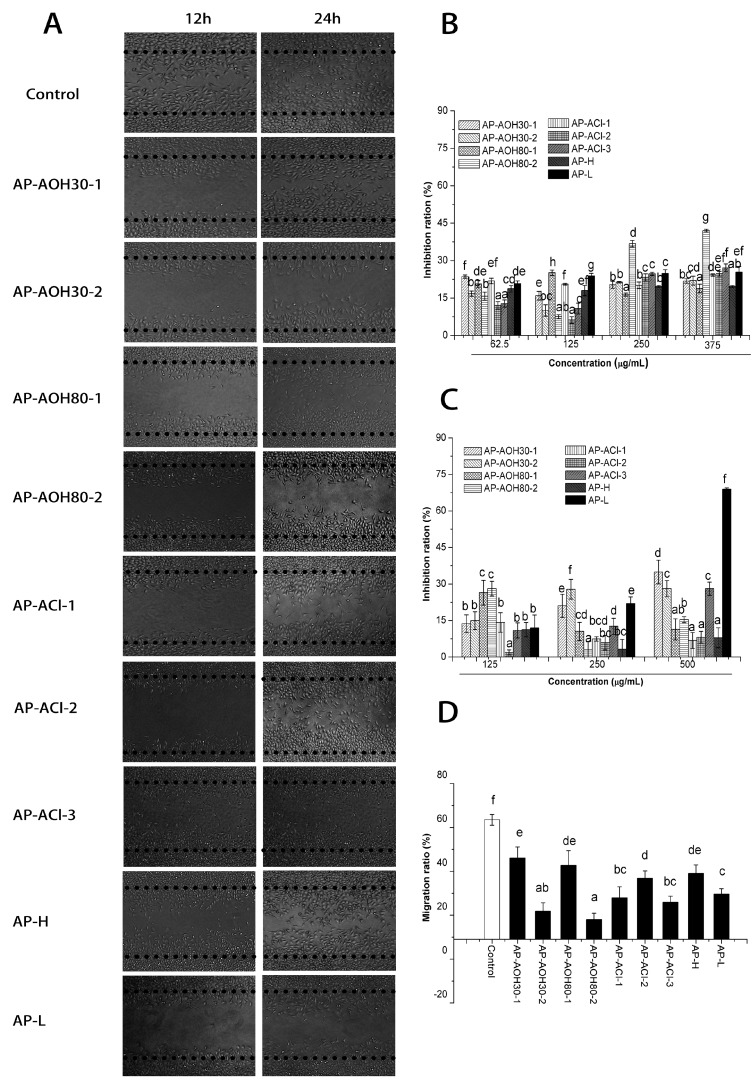
Anti-tumor effects of APF. (**A**) Images (100×) of the migration of APF-treated MCF-7 cells by wound healing assay; Antiproliferative activity of APF towards HepG2 cells (**B**) and towards MCF-7 cells (**C**); Cell migration rate of MCF-7 cells treated with APF after 24 h (**D**). The values are presented as the mean ± SD of triplicates. ^a–f^ in the column denote significant difference, mean values with different letters are significantly different (*p* < 0.05).

The inhibitory effects of the polysaccharide fractions on tumor cell lines (HepG2 cells and MCF-7 cells) are shown in Figure 2. Results demonstrate that great differences exist in the anti-proliferation activity between the APF. AP-L, AP-ACl-2 and AP-ACl-3 could inhibit the proliferation of HepG2 cells dose-dependently. The general level of anti-proliferation was 10%–20% for all fractions against HepG2 cells. Only AP-AOH80-2 showed higher anti-proliferation effects (above 30%) at the concentrations of 250 and 375 µg/mL.

In the MCF-7 anti-proliferation assay, Figure 2C shows that among the APF, the growth of MCF-7 cells could be inhibited by AP-L, AP-AOH30-1 and AP-AOH30-2 in a dose dependent manner. At the higher concentration of 375 µg/mL, the inhibition rate of AP-L reached to 69%, while other fractions showed a relatively low inhibition with inhibition rates less than 40%.

The effects on the proliferation of normal human liver cell line HL-7702 were also determined by MTT assy. The results of the experiment showed that all the APF had no toxic effect on HL-7702 cells compared with those of the blank control (*p* > 0.05) when the concentrations up to 1000 µg/mL.

#### 2.3.2. *In Vitro* Inhibitory Effects of APF on Migration of MCF-7 Cells

A wound healing assay was employed to investigate the effects of migration inhibition by the polysaccharide fractions. MCF-7 cell migration was attenuated after APF treatment for 24 h, shown in Figure 2A. Compared to 63.51% of for the control shown in Figure 2D, the migration rate of MCF-7 was greatly inhibited by AP-AOH80-2, AP-AOH30-2, AP-ACl-3 and AP-ACl-1 with the migration rate of 18.02%, 21.84% and 25.90%, respectively. Furthermore, the inhibition rate of MCF-7 proliferation induced by these three fractions was no more than 30% at the concentration of 250 µg/mL (shown in Figure 2C), suggesting that their effect on MCF-7 cells is primarily migration inhibition rather than a direct cytotoxic effect.

### 2.4. In Vitro Immunomodulatory Activities

#### 2.4.1. *In Vitro* Effect of APF on Lymphocyte Proliferation

**Figure 3:**
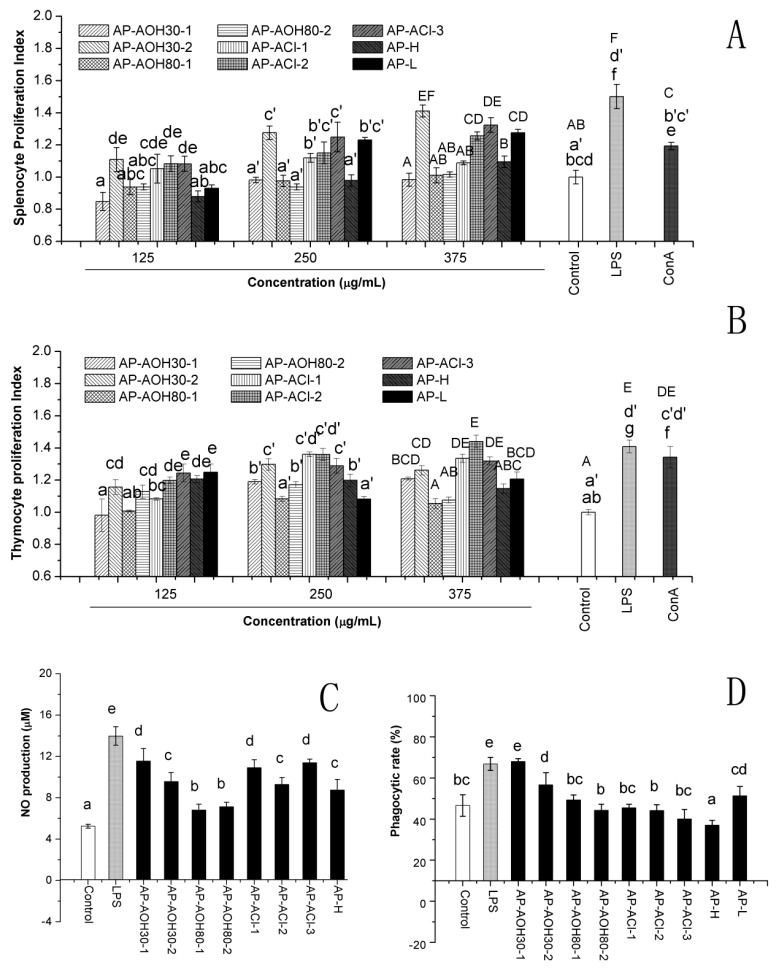
*In vitro* influences of APF on the splenocyte proliferation (**A**) and thymocyte proliferation (**B**), NO production (**C**) and phagocytic activity (**D**) of peritoneal macrophages. Data are expressed as the means ± SD. ^a–g, a’–d’, A–F^ Mean values in the column with different letters are significantly different (*p* < 0.05).

As shown in Figure 3A, stimulatory activity was different between the nine fractions. The splenocyte proliferative effects of AP-AOH30-2, AP-ACl-2, AP-ACl-3 and AP-L were significant (*p* < 0.05), compared to the blank control in almost all the tested concentrations, indicating that they all appeared to stimulate splenocyte proliferation. These stimulatory effects appeared to be dose-dependent manner, and their stimulating index at high concentration was even comparable to the positive control (LPS and ConA).

Significant differences (*p* < 0.05) among the nine fractions were also observed in the thymocyte proliferation assay as shown in Figure 3B. Fractions AP-AOH30-2, AP-ACl-1, AP-ACl-2 and AP-ACl-3 displayed significant (*p* < 0.05) thymocyte proliferative effects in all the tested concentrations as compared with the blank control, suggesting that they all could induce a facilitation of thymocyte proliferation. It was interesting to note that AP-AOH30-2, AP-ACl-2 and AP-ACl-3, which were more effective on the splenocyte and thymocyte proliferation, were all eluted by NaCl solution of different concentrations on the anion-exchange column, suggesting the splenocyte and thymocyte proliferation response might be associated with the charge property of polysaccharide.

#### 2.4.2. Nitric Oxide (NO) Production

Change in the NO production in macrophages co-culture with APF was investigated as shown in Figure 3C. All fractions could significantly induce the secretion of NO (*p* < 0.05) compared to untreated macrophages. The fraction AP-AOH30-1 displayed highest activity and AP-AOH30-2, AP-ACl-1 and AP-ACl-3 showed relatively higher activity to stimulate the NO release of macrophages.

#### 2.4.3. Peritoneal Macrophage-Mediated Cytotoxicity Assay

In order to explore the macrophage-activating potential of various polysaccharides, untreated and different APF or LPS-treated peritoneal macrophages were co-cultured with MCF-7 tumor cells to determine the cytotoxic activity of APF-activated macrophages. As shown in Figure 3D, AP-AOH30-1 and AP-AOH30-2 could significantly enhance the tumoricidal activity of the peritoneal macrophages as compared to untreated macrophages (*p* < 0.05), whereas, no significant tumoricidal activity was observed by the peritoneal macrophages primed with other polysaccharide fractions.

### 2.5. Comprehensive Comparison by Rank-Sum Ratio (RSR) Method

The RSR method was used to compare the differences in anti-tumor and immunomodulatory effects of APF. According to the RSR values as listed in Table 4, AP-AOH30-2, AP-ACl-3 and AP-L had relatively higher RSR_compr values of 0.6875, 0.6667 and 0.6875, respectively, indicating higher anti-tumor and immunomodulatory effects than other fractions. AP-L is seen to be the most active in anti-tumor effects with the RSR_anti values of 0.7778, whereas AP-AOH30-2 and AP-ACl-3 exhibit more potent effects in the immunomodulatory assay with RSR_immu values of 0.8194 and 0.7917, respectively. These results demonstrated that different isolated fractions exhibited complex behavior in the anti-tumor and immunomodulatory effects.

The possible factors affecting the observed bioactivities of *A. cantoniensis* polysaccharides were also investigated as shown in Table 3. The fractions APF_30%EtOH had higher RSR_anti, RSR_immu and RSR_compr values of 0.6250, 0.6389 and 0.6319, respectively, suggesting the bioactive polysaccharides could be precipitated by 30% ethanol. The APF_sepharose fractions had higher RSR_compr values of 0.5949 compared with 0.5313 for the APF_cellulose fractions, indicating that the bioactivity of polysaccharide can be affected by the method of anion exchange chromatography purification. The RSR_compr values of acidic polysaccharides (APF_acidic) was 0.5799, indicating slightly better bioactivities than neutral polysaccharides (APF_neutral) and this may be ascribed to their acidic properties.

## 3. Discussion

Polysaccharides have been found to be important bioactive compounds in a large number of natural resources, and have attracted a plethora of attention in the biochemistry and medical areas. The anti-tumor and immunomodulatory activities of polysaccharides are the most attractive for further work, owing to their low cytotoxicity to normal healthy cells and no obvious side effects in patients [7]. Nowadays, cancer prevention includes suppressing or inhibiting the development of cancer progression and metastasis, and the latter involves complex steps including cell adhesion, invasion and migration, which are the primary cause of mortality and relapse in cancer patients [16]. Therefore, anti-tumor effects including anti-proliferation and anti-migration were evaluated in this study. According to our results (Figure 2), *A. cantoniensis* polysaccharides showed good activity in preventing the migration of MCF-7 breast cancer cells. Some of the APF could also significantly suppress the proliferation of HepG2 cells and MCF-7 cells in a concentration-dependent manner. Interestingly, APF with the exception of AP-ACl-2, AP-ACl-3, AP-AOH80-2, AP-AOH30-1, AP-AOH30-2 and AP-L did not significantly inhibit the cellular proliferation of MCF-7 cells or HepG2 directly, suggesting that they could not significantly inhibit the proliferation of cancer cells *in vitro*. Similar results were also observed in polysaccharides from *Ganoderma atrum* [7], *Ampelopsis megalophylla* [17], *Actinidia eriantha* [18], *Andraspberry pulp* [19], which have been reported to exhibit no significant or lower anti-tumor effects *in vitro*. However, some of these polysaccharides such as *Ganoderma atrum* polysaccharide and *Ampelopsis megalophylla* polysaccharide have been shown to exhibit anti-tumor effects *in vivo* indirectly by enhancing the immune response. Therefore, we also investigated the immunomodulation activities of the nine polysaccharide fractions identified herein. Splenocyte and thymocyte proliferation is an indicator of immune enhancement, and they are macrophages that are highly secretory phagocytic cells and they play a vital role in host innate defense against any type of invasive cell including tumor cells [20]. According to the results in Figure 3, some APF could promote the proliferation of splenocytes and thymocytes, and enhance the NO secretion as well as showing cytotoxic effects towards MCF-7 cells of peritoneal macrophages.

Numerous studies have reported that the method of extraction and isolation can affect the activities of polysaccharides from various origins [21,22]. It is therefore important to determine suitable processing techniques in order to retain the bioactive polysaccharides. Among the various purification processes, ethanol precipitation, membrane separation and ion-exchange chromatography (IEX) are widely applied as routine purification processes for polysaccharides. Almost all of the reported polysaccharides have been fractionated by at least one or two of such these steps, although they are tedious and inefficient [23]. Thus, in this investigation, those three routes were applied for fractionating polysaccharides. The rank sum ratio (RSR) method was used to compare the differences in anti-tumor and immunomodulatory effects of APF obtained from different fractionation processes. The RSR method is widely used in comprehensive evaluation of multi-index, statistical forecasting and in statistical quality control in the medical and health fields; the larger the RSR value is, the more superior it is [24]. Based on this principle, the fraction having a higher RSR value indicates a more suitable bioactivity. Among the purified fractions, AP_AOH30-2 had highest RSR_compr value followed by AP-ACl-3. However, considering the higher yield and carbohydrate content of AP-ACl-3, AP-ACl-3 these fractions were selected for further study on both detained characterization and *in vitro* biological investigation.

Possible factors behind these differences were also explored as displayed in Table 3. Ethanol precipitation and membrane separation are widely used in producing natural extracts with the advantages of high throughput and ease of ability to scale up processing. In this study the polysaccharide in *A. cantoniensis* was greatly affected by the ethanol precipitating concentration and the 30% ethanol precipitate contained more bioactive fractions than the 80% ethanol precipitate. Additionally, molecular weight appears to be an important feature affecting the bioactivity of natural polysaccharides [25], thus a membrane technique was used to separate polysaccharides using a membrane with the molecular weight cut-off of 10 × 10^4^ Da. The molecular weight results in this study combined with bioactive values indicate those smaller polysaccharides around 4.4 × 10^4^ and 1.5 × 10^4^ Da seemed to be the most bioactive fractions of *A. cantoniensis* polysaccharide. Besides, fractions AP-AOH30-2 and AP-ACl-3, with higher RSR_compr values, also contained a relatively high content of uronic acid, indicating that the anti-tumor and immunomodulatory activity of polysaccharides might be associated with their anionic nature. This was further confirmed by comparing the bioactivities between neutral and acidic polysaccharides by RSR method. Similar results were also found in the polysaccharides from the fruiting bodies of *Zizyphusjujuba cv.* Jinsixiaozao, and fractions containing more uronic acid had stronger free radical scavenging activities than fractions with no uronic acid [22]. Inngjerdingen *et al.* [26] also reported that acidic polysaccharide fractions exhibited more effective complement fixation activity than its neutral fractions.

Anion-exchange chromatography is widely applied to separate polysaccharides based on their different ionic properties [27]. Unlike ethanol precipitation and membrane separation in simple mode, anion-exchange chromatography applied to purifying polysaccharides represents a great diversity. Extracted polysaccharides can be further purified onto diethylaminoethyl (DEAE)-exchange chromatography column initially equilibrated with different anion form such as Cl^−^ [28], OH^−^ [29], CH3COO^−^ [30] and phosphate [31] or a combination [32] and then eluted by increasing salt ions (typically Cl*^−^*) of the buffer. Moreover, various DEAE group substituted matrices were used for fractionating polysaccharides including DEAE-52 cellulose, DEAE-Sepharose fast flow, DEAE-Sepharose CL-Band DEAE-sephadex A-25 as well as ANX Sepharose 4 fast flow [26], which makes this purification process more complicated and diverse. Among the reported anion-exchange chromatography process for the isolation of polysaccharides, DEAE-52 cellulose and DEAE-Sepharose Fast Flow column converted to Cl^−^ or OH^−^ form, are the most widely used in fractionating polysaccharides from various origins. In a weak anion exchange system, when binding (or elution) occurs there is an immediate change to the ionic strength and pH, albeit a small change. For the APF_cellulose fractions, when the acidic polysaccharides bound to such an exchanger they initially would have to be converted to an OH^−^ form, OH^−^ ions would subsequently be lost, forming an alkaline environment. This study first demonstrated the initial anion form of the anion exchanger had great effect on the structural properties and bioactivity of polysaccharide.

In conclusion, this study demonstrated that the process used for their purification had great effect on the yield, chemical properties including sugar content, the content of uronic acid, molecular weight and monosaccharide composition, and the *in vitro* anti-tumor and immunomodulatory activities of the polysaccharides. Finally, AP-ACl-3 was considered to be more worthy of further study than other fractions on both the detailed structure characteristics and *in vivo* biological activities. AP-ACl-3 possessed anti-migration activities and immunomodulatory ability through facilitating the proliferation of lymphocytes and enhancing the NO secretion of peritoneal macrophages. Such information would facilitate the development of the polysaccharide in A. cantoniensis in food and pharmaceutical application.

## 4. Materials and Methods

### 4.1. Materials

*A. cantoniensis* was sliced and ground into powder using a milling machine. Male BALB/c mice of 8–10 weeks old (approval documents: SCXK/20110029) were purchased from the Experimental Animal Center of Sun Yat-sen University (Guangzhou, China). All the animal experiments were approved and carried out in accordance with relevant laws and the guidelines set by the Center of Animal Testing of Sun Yat-sen University.

### 4.2. Chemicals

DEAE-52 cellulose was purchased from Waterman (Maidstone, UK) and DEAE-sepharose fast flow was purchased from GE health care (Fairfield, CT, USA). Monosaccharide standards including glucose (Glc), xylose (Xyl), fructose (Fru), arabinose (Ara), galactose (Gal), fucose (Fuc), mannose (Man), galacturonic acid (GalA), glucuronic acid (GlcA), rhamnose (Rha) and inositol were purchased from Sigma Chemical Co. (St. Louis, MO, USA). 3-(4,5-Dimethylthiazol-2-yl)-2,5-diphenyltetrazolium bromide (MTT), lipopolysaccharide (LPS) and concanavalin A (ConA) were also bought from Sigma Chemical Company. WME medium, DMEM medium, Hank’s balanced salt solution (HBSS), 4-(2-hydroxyethyl)-1-piperazineethane-sulfonic acid (HEPES), insulin, penicillin, streptomycin and gentamicin were obtained from Gibco Biotechnology Co. (Grand Island, NY, USA). Chromogenic TAL/TAL endpoint assay kit was bought from the Chinese Horseshoe Crab Reagent Manufactory, Co., Ltd. (Xiamen, China). All the other reagents used were of analytical grade.

### 4.3. Preparation of Crude Polysaccharides from the A. cantoniensis

Dried powder of *A. cantoniensis* was extracted with 95% aqueous ethanol solution three times at 37 °C for 12 h until the extract became clear and colorless to remove pigments and small molecule compounds. Following centrifugation at 4500× *g* for 15 min, the resulting residues were dried at 60 °C for 12 h and extracted with deionised water in a ratio of 1:30 (*w*/*v*) for 3 h at 92 °C three times. After centrifugation at 4500× *g* for 15 min, the supernatants were combined and concentrated by rotary vacuum evaporator at 45 °C. Then the supernatants were added to anhydrous ethanol giving a final ethanol concentration of 80% (*v*/*v*) and kept at 4 °C overnight. The resulting precipitate was dissolved in deionised water and then centrifuged for 10 min at 8000× *g* to remove insoluble substances. The supernatant was dialyzed, concentrated and freeze-dried to obtain a crude *A. cantoniensis* polysaccharide (AP).

### 4.4. Fractionation and Isolation of A. cantoniensis Polysaccharide

#### 4.4.1. Fractionation of AP by Membrane Separation

The membrane separation was performed with a Millipore ultrafiltration system (Millipore, USA) using a membrane of 100 kDa molecular weight cut-off as shown in Figure 1. The retentate and permeate were collected separately, with the retentate being recirculated into the feed until maximum permeate yield was achieved. Permeate from the 100 kDa membrane was collected and dialyzed using a membrane dialysis tube (Spectrum; cut-off: 3.5 kDa). Finally, two fractions of polysaccharides, AP-H (>100 kDa) and AP-L (3.5–100 kDa), were obtained, concentrated and freeze-dried for further study.

#### 4.4.2. Fractionation of AP by DEAE-52 Cellulose Column Combined with Ethanol Stepwise Precipitation

The AP was dissolved in deionised water then centrifuged at 8000× *g* for 15 min. The resulting solution was collected and precipitated with ethanol at a final concentration of 30% (*v*/*v*) and the precipitate (AP-AOH30) was collected after the centrifugation at 4500× *g* for 15min. The supernatant was added ethanol to final concentration of 80% (*v*/*v*) to obtain another precipitate (AP-AOH80). The precipitates AP-AOH30 and AP-AOH80 were further subjected to a DEAE-52 cellulose column (2.6 × 50 cm, OH^−^ form), which was eluted with deionised water and 0.2 M NaCl solution. The elution solutions were collected, concentrated, dialyzed (cut-off *M*_W_: 3.5 kDa) and lyophilized to obtain four fractions (AP-AOH30-1, AP-AOH30-2, AP-AOH80-1 and AP-AOH80-2).

#### 4.4.3. Fractionation of AP by DEAE-Sepharose Fast Flow Column

The AP solution was applied to a DEAE-sepharose fast flow column (2.6 × 50cm, Cl^−^ form) directly, followed by stepwise elution with 0.0, 0.2, 0.5 M NaCl solutions. The fractions in each peak were dialyzed and freeze-dried, resulting in three polysaccharide fractions (AP-ACl-1, AP-ACl-2 and AP-ACl-3). The fractionating steps of all the *A. cantoniensis* polysaccharide fractions (APF) were shown in Figure 1.

### 4.5. Characterization Comparison for APF

#### 4.5.1. Chemical Composition of APF

The carbohydrate, protein, uronic acid, and sulfate content of polysaccharide fractions were respectively measured by phenol-sulfuric acid method [33], Bradford method [34], *m*-hydroxydiphenyl method [35] and barium chloride-gelatin method [36] respectively. Possible endotoxin contamination was analyzed using a chromogenic TAL/TAL endpoint assay kit with the detection limit of 0.005–1 EU/mL as described previously [37].

#### 4.5.2. Determination of Molecular Weight

The molecular weight of APF was determined by an Agilent 1260 HPLC system equipped with a refractive index detector (RID). The separation was completed on a TSK-GEL G3000PWxl column (7.8 × 300 mm; Tosoh, Tokyo, Japan) coupled with a TSK-GEL G5000PWxl column (7.8 × 300 mm; Tosoh) according to the method explained previously [38].

#### 4.5.3. FT-IR Spectral Analysis

The FT-IR spectrum was recorded with a Vector 33 FT-IR spectrophotometer (Bruker, Ettlingen, Germany) as reported by Xu *et al.* [39]. The dried polysaccharides were ground and pressed into potassium bromide (KBr) pellets for measurement in the wave number range of 4000–400 cm^−1^.

#### 4.5.4. Determination of Monosaccharide Composition

Polysaccharides (5 mg) were hydrolyzed with 4 mol/L trifluoroacetic acid (2 mL) at 110 °C for 5 h and then the hydrolysate was concentrated by a stream of nitrogen at 70 °C. The hydrolysates were derivatized through acetylation and the resulting alditol acetates were detected by gas chromatography (GC) according the method described previously [40].

### 4.6. Cell Line Cultures

Human liver cancer line HepG2 (ATCC HB-8065) and human breast cancer line MCF-7 (ATCC HTB-22) and normal human liver cell line HL-7702 were purchased from ATCC company. HepG2 cells were cultured in growth medium (WME supplemented with 5% fatal bovine serum (FBS), 10 mM Hepes, 2 mM l-glutamine, 5 µg/mL insulin, 0.05 µg/mL hydrocortisone, 50 units/mL penicillin, 50 µg/mL streptomycin, and 100 µg/mL gentamycin). MCF-7 cells and HL-7702 cells were cultured in DMEM supplemented with 10% (*v*/*v*) FBS and 50 units/mL of penicillin and 50 µg/mL of streptomycin and 100 µg/mL gentamycin. HepG2 and MCF-7 cells were maintained at 37 °C in a humidified atmosphere containing 5% CO_2_.

### 4.7. Anti-Tumor Assay

#### 4.7.1. Antiproliferation Activity

APF at various concentrations (62.5–375 µg/mL) were incubated with HepG2 cells and MCF-7 cells for 48 h at 37 °C, respectively. The blank control was treated with the fresh medium alone. Then the effects of APF on the proliferation of HepG2 cells and MCF-7 cells were determined by MTT method as reported elsewhere [41]. The proliferation inhibition rate was calculated using the formula:

(1) $$\text{Inhibition}\left(\%\right)=\left(1-Abs_{2}/Abs_{1}\right)\times 100$$

where *Abs*_1_ is the absorbance of control, and *Abs*_2_ is the absorbance of treated cells.

#### 4.7.2. Wound Healing Assay

Cell migration assay was performed as described by Park *et al.* [42]. Briefly, after the wounded MCF-7 cells monolayer was ready, 500 µL of fresh medium with or without APF (250 µg/mL) were incubated with the wounded cell monolayer. The wound area were observed and captured by a microscope and cell migration rate was calculated by Image J software version 1.48v [42]. The anti-migration rate was presented as the percentage of cell covering area to the wounded area of corresponding 0 h.

### 4.8. Immunomodulatory Activities

#### 4.8.1. Splenocyte and Thymocyte Proliferation Test

Splenocytes and thymocytes’ isolation and culture was carried out as reported before [43]. The cells were washed with PBS, adjusted to a density of 5 × 10^6^ cells/mL in DMEM containing 10% FBS (*v*/*v*) and kept on ice for further use. A spleen or thymus lymphocyte proliferation assay was implemented according to the previous method [40]. Briefly, a splenocyte or thymocyte suspension was incubated with or without APF (125, 250 and 375 µg/mL). Wells containing ConA (final concentration of 5 µg/mL) or LPS (final concentration of 10 µg/mL) alone served as positive control and with medium alone as blank control. After the cells were cultured at 37 °C for 48 h, the splenocyte proliferation index or thymocyte proliferation index was determined by MTT method and calculated from the ratio of the optical density (OD) values of treated group to the control group.

#### 4.8.2. Determination of Nitric Oxide (NO) Production

Peritoneal macrophages were obtained from the peritoneal cavity of mice as described earlier [44]. Non-adherent cells were removed by washing with medium and adherent macrophages were further cultured with fresh medium alone or media containing APF (250 µg/mL) for 48 h. Cells treated with LPS (10 µg/mL) served as positive control. At the end of the culture period, nitrite content (NO) secreted by peritoneal macrophage was determined by a NO assay kit (Nanjing Jiancheng Bioengineering Institute, Nanjing, China) according to the manufacturer’s instruction [45].

#### 4.8.3. Mouse Peritoneal Macrophage-Mediated Cytotoxicity

The assay for macrophage-mediated cytotoxicity was measured according to the method described elsewhere with some modification [46,47]. Briefly, the mouse peritoneal macrophages (2 × 10^6^ cells/mL) were seeded in 96 well plates and allowed to adhere for 2 h at 37 °C in 5% CO_2_ humidified incubator. Non-adherent cells were removed by washing with medium and adherent macrophages were further cultured with fresh medium alone, or different APF at the concentration of 250 µg/mL or LPS (10 µg/mL, as positive control) for 48 h at 37 °C. The macrophages were washed with DMEM medium to remove the stimulants and co-incubated with MCF-7 cells for 24 h with the ratio of macrophage to target cells was approximately 40:1. The percentage of growth inhibition of MCF-7 cells was determined by using the MTT method. Cytolytic activity was expressed as the percentage of tumor cytotoxicity as follows:

(2) %Cytotoxicity={1−O.D.if[(target+macrophages)−macrophages]/O.D.of[target(unreated)]}×100

### 4.9. Rank Sum Ratio Analysis

Rank sum ratio analysis was conducted on the anti-tumor and immunomodulatory effects of APF to determine a comprehensive index of results as reported elsewhere [24]. Code ranking was allocated for each object with the greater priority having the higher value. RSR value was calculated by following formula:

(3) $$\text{RSR}=\frac{\sum_{j=1}^{m}R_{ij}}{mn}$$

where, *n* represented the number of objects and m represented the number of indices; where, *i* = 1, 2…, *n*; j = 1, 2…, *m*; R*_ij_* is the element in row *i* and column *j*.

### 4.10. Statistical Analysis

Data is expressed as means ± standard deviations (SD) (*n* = 3) of three replicates. One-way analysis of variance (ANOVA) was used to determine the significance of differences between the treatment groups and *p* values of 0.05 or less were considered to be statistically significant.

## Figures and Tables

**Table 1:** Chemical properties of Abrus polysaccharides fractions (APF).

Items	Yield (mg/kg)	Total Sugar ^1^ (*w*/*w*, %)	Uronic Acid ^1^ (*w*/*w*, %)	Sulfate ^1^ (*w*/*w*, %)	Distribution of Molecular Weight (×10^4^ Da)			
					Peak 1	Content (%)	Peak 2	Content (%)
AP-AOH30-1	237.1	74.28 ± 1.08 e	20.33 ± 1.70 f	8.48 ± 0.43 e	11.27	62.27	1.69	15.97
AP-AOH30-2	64.8	67.51 ± 1.91 cd	22.57 ± 1.48 g	11.05 ± 0.40 f	12.98	59.77	1.67	34.55
AP-AOH80-1	301.6	71.08 ± 1.67 de	1.84 ± 0.92 a	0.83 ± 0.77 a	5.73	14.96	0.51	78.93
AP-AOH80-2	93.7	66.97 ± 4.21 c	0.75 ± 0.30 a	0.98 ± 0.31 ab	2.61	85.16	n.d.^2^	n.d.
AP-ACl-1	702.3	82.19 ± 2.36 f	6.05 ± 0.38 b	1.93 ± 0.38 c	2.68	84.91	n.d.	n.d.
AP-ACl-2	504.5	84.78 ± 2.28 f	10.87 ± 2.01 d	1.62 ± 0.25 bc	14.05	52.06	1.97	22.36
AP-ACl-3	613.5	83.93 ± 1.60 f	13.62 ± 0.57 e	3.58 ± 0.16 d	4.4	83.31	n.d.	n.d.
AP-H	865.2	55.78 ± 1.18 a	2.52 ± 1.08 a	1.06 ± 0.29 ab	8.54	36.45	5.44	53.20
AP-L	121.4	59.93 ± 1.54 b	9.31 ± 1.03 c	3.88 ± 0.32 d	1.45	75.57	n.d.	n.d.

^1^ Average of duplicate analyses ± standard deviation; symbols bearing different letters (a–g) in the same column with each treatment are significantly different (*p* < 0.05); ^2^ Not detected.

**Table 2:** Wavelength assignments (cm^−1^) for the IR bands of APF.

Items	AP-AOH30-1	AP-AOH30-2	AP-AOH80-1	AP-AOH80-2	AP-ACl-1	AP-ACl-2	AP-ACl-3	AP-H	AP-L	Assignments ^2^
Wavelength (cm^−1^)	3435	3425	3421	3440	3435	3429	3431	3426	3656	v O–H
	2927	2934	2934	2933	2942	2930	2925	2939	2941	v C–H
	1743	1743	n.d.^1^	n.d.	n.d.	1735	1723	n.d.	1739	δ C=O
	1421	1420	n.d.	n.d.	n.d.	1424	1424	n.d.	1415	δ O–C=O
	1371	1373	1238	1334	1334	1373	1370	1334	1367	Sulpates
	1240	1240	n.d.	1236	1236	1241	1240	1236	1241	v O=S=O
	578	579	n.d.	587	n.d.	578	576	585	n.d.	δ O=S=O
	895	894	893	896	894	898	895	893	894	β-linked
	850	854	n.d.	853	854	n.d.	857	n.d.	855	α-linked

^1^ Not detected; ^2^ Refer to the references of [14,15]; v. stretching vibration; δ. bending vibration.

**Table 3:** Monosaccharide composition of APF.

Fractions	Sugar Components (%)							
	GlcA	GalA	Rha	Ara	Xyl	Fuc	Glc	Gal
AP-AOH30-1	n.d.^1^	10.0	7.4	26.2	n.d.	8.1	9.0	39.3
AP-AOH30-2	46.1	22.1	4.0	11.7	n.d.	n.d.	3.5	12.6
AP-AOH80-1	n.d.	n.d.	n.d.	8.8	2.9	3.2	69.6	15.5
AP-AOH80-2	n.d.	2.1	15.1	18.1	2.7	9.4	6.2	46.5
AP-ACl-1	n.d.	n.d.	n.d.	n.d.	n.d.	n.d.	83.7	16.3
AP-ACl-2	n.d.	5.5	3.5	6.3	2.9	3.4	64.8	13.6
AP-ACl-3	61.1	3.0	9.8	8.9	n.d.	3.0	4.3	9.9
AP-H	n.d.	n.d.	n.d.	30.9	n.d.	n.d.	12.3	56.8
AP-L	n.d.	7.6	9.1	17.0	3.0	5.2	30.3	27.7

^1^ Not detected.

**Table 4:** Differences in the anti-tumor and immunomodulatory effects of APF and the underlying affecting factors assessed by rank-sum ratio (RSR) method.

Items	RSR_anti ^1^	RSR_immu	RSR_compr
AP-AOH30-1	0.6944	0.4583	0.5764 ^5^
AP-AOH30-2	0.5556	0.8194	0.6875
AP-AOH80-1	0.5833	0.2778	0.4306
AP-AOH80-2	0.5417	0.3194	0.4306
AP-ACl-1	0.5278	0.6389	0.5833
AP-ACl-2	0.3750	0.6944	0.5347
AP-ACl-3	0.5417	0.7917	0.6667
AP-H	0.4028	0.3611	0.3819
AP-L	0.7778	0.5972	0.6875
APF_30%EtOH ^2^	0.6250	0.6389	0.6319
APF_80%EtOH	0.5625	0.2986	0.4306
APF_sepharosec ^3^	0.4815	0.7083	0.5949
APF_cellulose	0.5938	0.4687	0.5313
APF_neutrald ^4^	0.6018	0.3148	0.5301
APF_acidic	0.5034	0.6563	0.5799

^1^ RSR_anti, RSR_immun, RSR_compr are calculated by RSR method from the anti-tumor assay, immunomodulatory assay and all the biological assessments, respectively; ^2^ APF_30%EtOH and APF_80%EtOH are all the polysaccharides precipitated by 30% and 80% (*v*/*v*) ethanol, respectively; ^3^ APF_sepharose and APF_celluose are all the polysaccharides fractionated by DEAE-sepharose fast flow (Cl^−^ form) column and DEAE-52 cellulose (OH^−^ form) column, respectively; ^4^ APF_neutral and APF_acidic are all the polysaccharides eluated by water and NaCl solution, respectively; ^5^ The larger of the RSR value is the more superior it is.

## Supplementary Materials

Supplementary materials can be found at http://www.mdpi.com/1422-0067/17/4/511/s1.
