# Supplementary material for: The Effects of Different Purifying Methods on the Chemical Properties, in Vitro Anti-Tumor and Immunomodulatory Activities of Abrus cantoniensis Polysaccharide Fractions
Source: Int J Mol Sci. 2016 Apr 6;17(4):511. doi: 10.3390/ijms17040511 (PMC4848967; doi:10.3390/ijms17040511)
Supplement: Supplementary file 1 [file ijms-17-00511-s001.pdf]

Supplementary Materials: The Effects of Different Purifying Methods on the Chemical Properties, *in Vitro* Anti-Tumor and Immunomodulatory Activities of *Abrus cantoniensis* Polysaccharide Fractions

Shaowei Wu, Xiong Fu, Margaret A. Brennan, Charles S. Brennan and Chen Chun

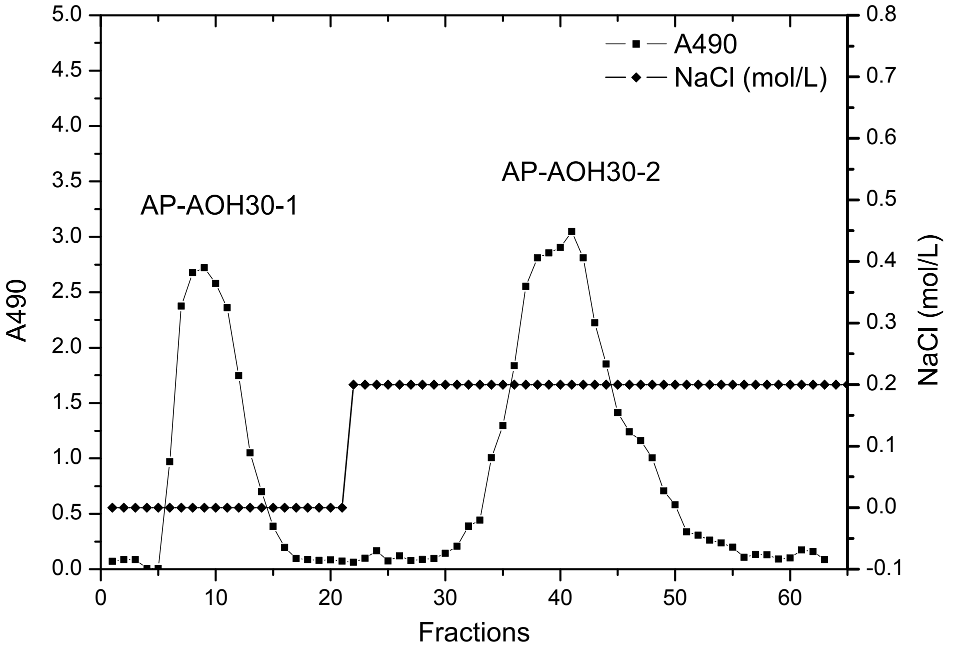

Figure S1. Elution curve of crude polysaccharide AP-AOH30 purified by DEAE-52 cellulose anion-exchange chromatography.

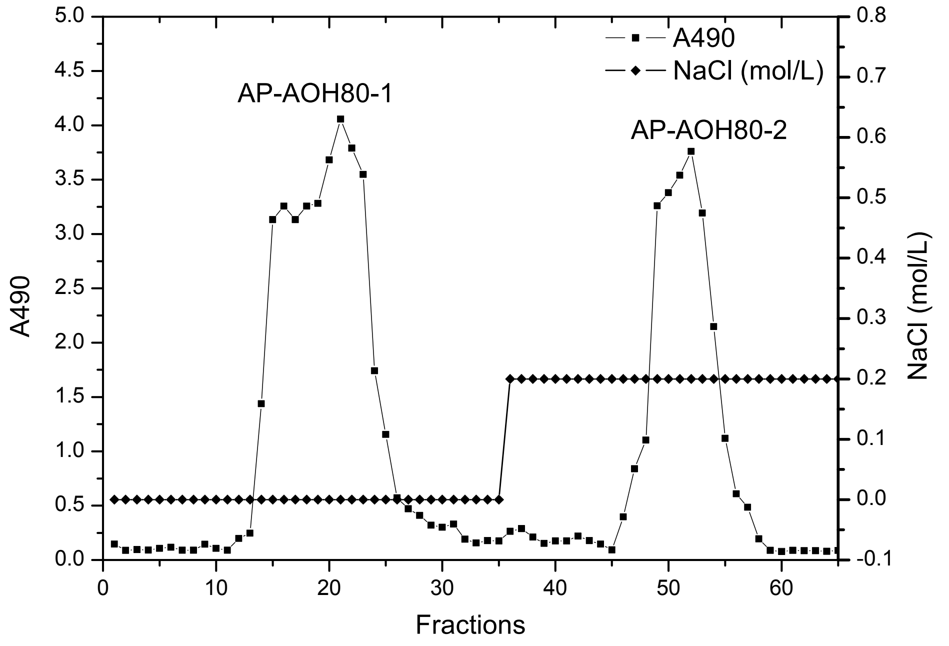

Figure S2. Elution curve of crude polysaccharide AP-AOH80 purified by DEAE-52 cellulose anion-exchange chromatography.

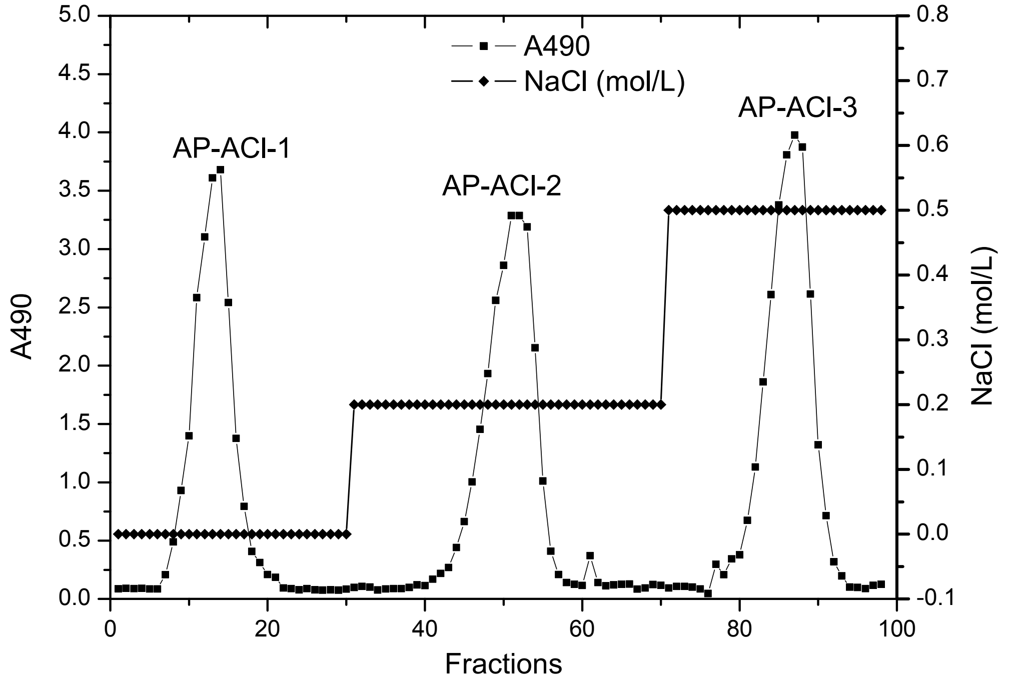

Figure S3. Elution curve of crude polysaccharide AP purified on the DEAE-sepharose fast flow column.
